# Real-world data of cardio-oncologic interventions for cardiovascular adverse events with oral oncolytics

**DOI:** 10.1186/s40959-024-00221-5

**Published:** 2024-04-09

**Authors:** Karen Abboud, Godsfavour Umoru, Barry Trachtenberg, Veronica Ajewole

**Affiliations:** 1 Department of Pharmacy, Houston Methodist Hospital, Houston, TX USA; 2 Heart Failure and Transplantation Cardiology, Houston Methodist Hospital, Houston, TX USA; 3 Texas Southern University College of Pharmacy & Health Sciences, Houston, TX USA

**Keywords:** Oral oncolytics, Cardiovascular toxicity, Cardiovascular adverse events, Cardio-oncology

## Abstract

**Background:**

Oral cancer therapy-related cardiovascular (CV) toxicity has a wide variety of presentations including arrhythmia, cardiomyopathy, and myocardial infarction, but clinical evidence related to its management is limited. The purpose of this IRB-approved, single-center, retrospective, cohort study was to characterize cardio-oncologic interventions for CV adverse events related to oral oncolytics.

**Methods:**

The cohort included 67 patients who were admitted to a multi-hospital health system between June 1, 2016 and July 31, 2021, had at least one medical record order of oral oncolytics considered to have cardiotoxic potential, and had an ICD10 code for a cardiotoxic event added to their electronic medical records after initiation of oral oncolytics.

**Results:**

The majority (97%) had pre-existing cardiovascular disease (CVD) or a CV risk factor. The three most common classes of oral oncolytics were aromatase inhibitors (36%), BCR-ABL inhibitors (16%), and VEGFR inhibitors (13%). New-onset or worsening heart failure (HF) (*n* = 31), which occurred after a median of 148 days (Interquartile range (IQR) 43–476 days) was the most common cardiotoxic event. The most frequent interventions were pharmacological treatment of the CV adverse event (*n* = 44) and treatment interruption (*n* = 18), but guideline-directed medication therapy for HF could be further optimized.

**Conclusion:**

Pre-existing CVD or CV risk factors predispose oncology patients to CV adverse events. Real-world practice reveals that CV adverse events require temporary interruption of treatment and initiation of pharmacologic treatment. A multidisciplinary, patient-centered approach that includes discussion of risks/benefits of treatment continuation, and initiation of guideline-directed treatment is recommended until high-quality, drug-specific data for monitoring and treatment become available.

## Background

As research continues to elucidate the cellular signaling pathways responsible for malignant cell transformation, the development of oral oncolytics in recent years has exponentially increased. Between March 2009 and May 2022, the US Food and Drug Administration (FDA) approved approximately 100 oral oncolytics with some agents gaining multiple indications [1]. The advent of oral oncolytics has dramatically improved overall cancer survival rates in several cancer types such as chronic lymphocytic leukemia, chronic myeloid leukemia, non-small cell lung cancer, hepatocellular cancer, and renal cell carcinoma and has led to increased attention to survivorship care and the potentially deleterious adverse effects that these medications may have [2–8]. The concern for oral oncolytics’ toxicities is compounded by the fact that these medications may be erroneously perceived as safer than parenteral antineoplastic agents, and most centers do not have a standardized, consistent process for follow up and monitoring of adverse effects [9–13]. Several oral oncolytics, such as tyrosine kinase inhibitors (TKIs), target cellular signaling pathways that are implicated in oncogenesis but are also essential for normal physiological function of some organs, including the cardiovascular (CV) system [2]. CV toxicities are of particular concern since CV adverse events are underestimated in oncology trials due to the lack of standardized protocols for assessment of such events. Additionally, premarketing oncology trials include a small, strictly defined patient population with a low cardiovascular CV risk profile over a short duration of follow-up time, although oral oncolytics are usually given until disease progression or unacceptable toxicity, and as such, may not accurately ascertain the true incidence of these adverse events in a real-world population [14]. CV toxicities have been reported with increasing frequency in post-marketing surveillance and may compromise the success of effective antitumor treatment by adversely affecting the patients’ quality of life and overall survival [15]. Cardiotoxicity may arise from direct effects of cancer treatment on the heart’s function and structure. On the other hand, cancer patients often have underlying CV risk factors and comorbidities, and oral oncolytics may accelerate the development of cardiovascular disease (CVD) in this susceptible population, a phenomenon which has been termed the “two-hit hypothesis” [16, 17]. 

CV toxicities can have a wide variety of manifestations. Cancer-therapy-related cardiac dysfunction (CTRCD) ranges from asymptomatic decrease in the left ventricular ejection fraction (LVEF) to overt heart failure (HF), which is the most debilitating manifestation of CV toxicity [18]. CTRCD is most commonly defined as a decrease in LVEF over 10% points to a value below the lower limit of normal or a relative decrease in global longitudinal strain (GLS) of over 15% from baseline [18–20]. Asymptomatic CTCRD can be further stratified into mild (LVEF ≥ 50% along with a new relative decline in GLS by > 15% from baseline and/or new rise in cardiac biomarkers), moderate (new LVEF decline to 40–49%), or severe (new LVEF decline below 40%) [20]. CTRCD, specifically cardiomyopathy secondary to doxorubicin, has been associated with a 3.5-fold increased mortality risk and overall poorer prognosis compared with other types of cardiomyopathies [21]. Oral oncolytics with documented CTRCD include multitargeted TKIs, epidermal growth factor receptor (EGFR) / human epidermal growth factor receptor 2 (HER2) inhibitors, breakpoint cluster region-Abelson murine leukemia (BCR-ABL) inhibitors, immunomodulators, and antiandrogens [2]. Other CV toxicities include acute coronary syndromes (ACS), arrhythmias, pericardial disease, and venous thromboembolism and are defined similarly as in in the general population [2, 20]. 

Although a few consensus documents and position articles regarding monitoring and management of CV toxicity have been published, the lack of defined CV endpoints in clinical trials, low level of evidence, and heterogeneity in recommendations have complicated applying a practical approach in clinical practice, especially in relation to oral oncolytics [18, 20, 22, 23]. Most notably, the 2022 European Society of Cardiology (ESC) cardio-oncology guideline has been criticized for making a substantial number of class I recommendations with low level of evidence [20]. Out of 156 (57%) Class I recommendations, 118 (76%) were supported by the lowest evidence grade of C [24–26]. As patients receiving oral oncolytics are not required to present to an infusion center or hospital to receive treatment, follow-up monitoring approaches including cardiac biomarkers and cardiac imaging need to be redefined and tailored to the oral regimen. In addition, it remains unclear whether the oral oncolytic dose should be temporarily withheld or reduced, treatment should be discontinued, or the oral agent should be continued with initiation of cardioprotective medications. Given that the use of oral oncolytics is projected to rise, continued real-world studies and registries to address existing literature gaps and determine the best monitoring and treatment strategies for any of these agents with cardiotoxic potential are crucial. Thus, the purpose of this study is to describe real-world cardio- oncologic interventions for patients who have experienced CV adverse events after initiating oral oncolytics.

## Methods

### Study design

The hospital institutional review board (IRB) approved the study protocol with a waiver for informed consent. A retrospective chart review of electronic medical records (EMR) was conducted in a large academic metropolitan hospital system, comprised of seven community hospitals, between June 2016 and July 31, 2021.

### Study population

The study included patients who had at least one medical record order of oral oncolytics, excluding cytotoxic chemotherapy medications, considered to have a cardiotoxic potential based on prescribing information, supporting literature, or reported cases to the FDA Event Reporting System (FAERS) Pharmacovigilance Database, the FDA’s database for post-marketing safety surveillance. The cohort included patients who had an ICD-10 code for arrythmias, HF or cardiomyopathy, myocardial infarction, pericardial disease, and venous/and or arterial thrombosis added to their EMR after the start of oral oncolytics. Patients who were receiving oral oncolytics for non-oncologic indications and patients with unavailable progress notes on their EMR were excluded.

### Outcome measures

The primary endpoint was the characterization of cardio-oncologic and medication-related interventions such as dose reduction, treatment interruption, treatment discontinuation, and initiation of cardioprotective medications. Key secondary endpoints included the correlation between CV risk factors and observed incidence of CV toxicities, onset of CV adverse events in relation to initiation and duration of therapy, and identification of monitoring practices.

### Data Collection

All data collected with regards to patient characteristics, past medical history, oncologic history, specific oral oncolytics, duration of therapy, cardiotoxic manifestations, laboratory values, imaging results, and home medications were obtained via manual chart review. Owing to the frequency of aromatase inhibitors (AIs) identified during chart review, FAERS was queried on May 31, 2022 for “cardiac failure”, “cardiac failure congestive”, “cardiac failure chronic”, “cardiac failure acute”, “left ventricular failure”, “right ventricular failure”, “ventricular dysfunction”, “left ventricular dysfunction”, “cardiac dysfunction”, “systolic dysfunction”, “cardiac ventricular disorder”, “left ventricular enlargement”, “ventricular hypertrophy”, “left ventricular hypertrophy”, “cardiac hypertrophy”, “cardiomyopathy”, “hypertrophic cardiomyopathy”, “restrictive cardiomyopathy”, “toxic cardiomyopathy”, “ejection fraction decreased”, “ejection fraction abnormal”, “cardiac output decreased”, and “cardiac index decreased” secondary to AIs, namely “anastrozole”, “exemestane”, and “letrozole”, and all other drugs. Adverse events from FAERS were obtained from January 1, 1996 to March 31, 2022. All terms were grouped together for analysis.

### Statistical analysis

Baseline demographic, clinical, and laboratory data as well as primary and secondary endpoints were reported using descriptive statistics. Continuous variables were presented as mean, median, and interquartile range. Categorical values were expressed as absolute frequencies. To compare the risk of cardiotoxic events reported with AIs versus other drugs in the database, a signal disproportionality analysis was calculated by using the reporting odds ratio (ROR). The precision of the ROR was determined by 95% confidence intervals (95% CI). P-values were calculated by using chi-squared or Fisher’s exact test, and a p-value < 0.05 was considered statistically significant.

## Results

**Fig. 1:**
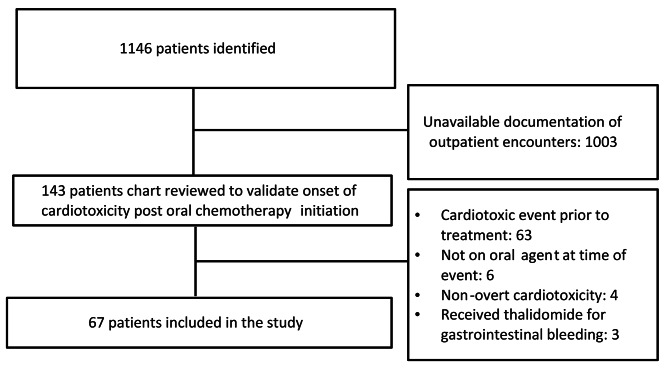
Flowchart showing derivation of the study cohort

A total of 1146 patients were identified to have an ICD-10 code for a cardiotoxic event added to their EMR after initiating oral oncolytics. Following exclusion of patients with unavailable documentation on their EMR, the charts of 143 were reviewed to validate onset of CV adverse events after initiation of oral oncolytics. After accounting for exclusion criteria, 67 patients were included in the final analysis (Fig. 1). Baseline characteristics were summarized in Table 1. The mean age was 69 ± 15 years and mean body-mass index (BMI) was 28.34 ± 6.73 kg/m2. The majority of patients were female (*n* = 45; 67.2%). Forty-five patients (67.2%) had solid tumors while 22 patients (32.8%) had hematologic malignancies. The most common single diagnosis was breast cancer (*n* = 26; 38.8%). Preexisting CV risk factors were prevalent in the cohort as 97% had at least one CV risk factor or established CVD. The most common baseline comorbidities were hypertension (*n* = 48; 71.6%), hyperlipidemia (*n* = 27; 40.3%), and obesity (*n* = 26; 38.8%). Prior chest radiation, anthracyclines, or trastuzumab was documented in 25.4%, 22.4% and 4.5% of patients, respectively. AIs (*n* = 25;46%), BCR-ABL inhibitors (*n* = 11;16%), and vascular endothelial growth factor receptor (VEGFR) inhibitors (*n* = 9;13%) were the three principal classes of oral oncolytics associated with CV adverse events.

**Table 1:** Baseline Characteristics for Evaluable Patients (*N* = 67)

Mean age, years (SD)	69 (± 15)
**Mean body-mass index, kg/m** ^**2**^ **(SD)**	28.34 (± 6.73)
**Gender (%)**	
Female	45 (67.2)
Male	22 (32.8)
**Race (%)**	
Caucasian	44 (65.7)
African American	18 (26.9)
Asian	3 (4.5)
Other	2 (3)
**Smoking status (%)**	
Never smoker	43 (64.2)
Former smoker	21 (31.3)
Current smoker	3 (4.5)
**Alcohol use (%)**	
No alcohol use	38 (56.7)
Infrequent to light use	21 (31.3)
Moderate use	7 (10.4)
Heavy use	1 (1.5)
**Cancer Diagnosis (%)**	
Breast cancer	26 (38.8)
Hematological malignancies	22 (32.8)
Other solid tumors	19 (28.4)
**Past Medical History (%)**	
Hypertension	48 (71.6)
Hyperlipidemia	27 (40.3)
Obesity	26 (38.8)
Diabetes	20 (29.9)
Arrhythmias	20 (29.9)
Heart failure (HF)	17 (25.3)
Coronary artery disease (CAD)	14 (20.9)
**Treatment History (%)**	
History of chest radiation	17 (25.4)
History of anthracyclines	15 (22.4)
History of trastuzumab	3 (4.5)
**Baseline Cardiovascular Disease (CVD) or CVD Risk Factor (%)**	
At least one risk factor	65 (97)
None	2 (3)
**Classification of Oral Oncolytics (%)**	
Aromatase inhibitors	25 (36)
BCR-ABL inhibitors	11 (16)
VEGFR inhibitors	9 (13)
Immuno-modulators	7 (10)
Antiandrogens	5 (7)
EGFR inhibitors	4 (6)
mTOR inhibitors	4 (6)
BTK inhibitors	3 (4)
FLT3 inhibitors	1 (2)

**Fig. 2:**
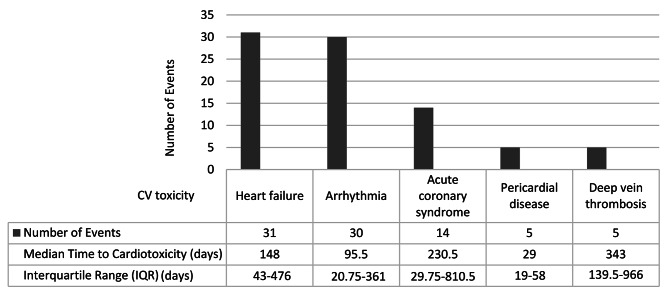
Incidence and classification of cardiovascular (CV) toxicity

**Fig. 3:**
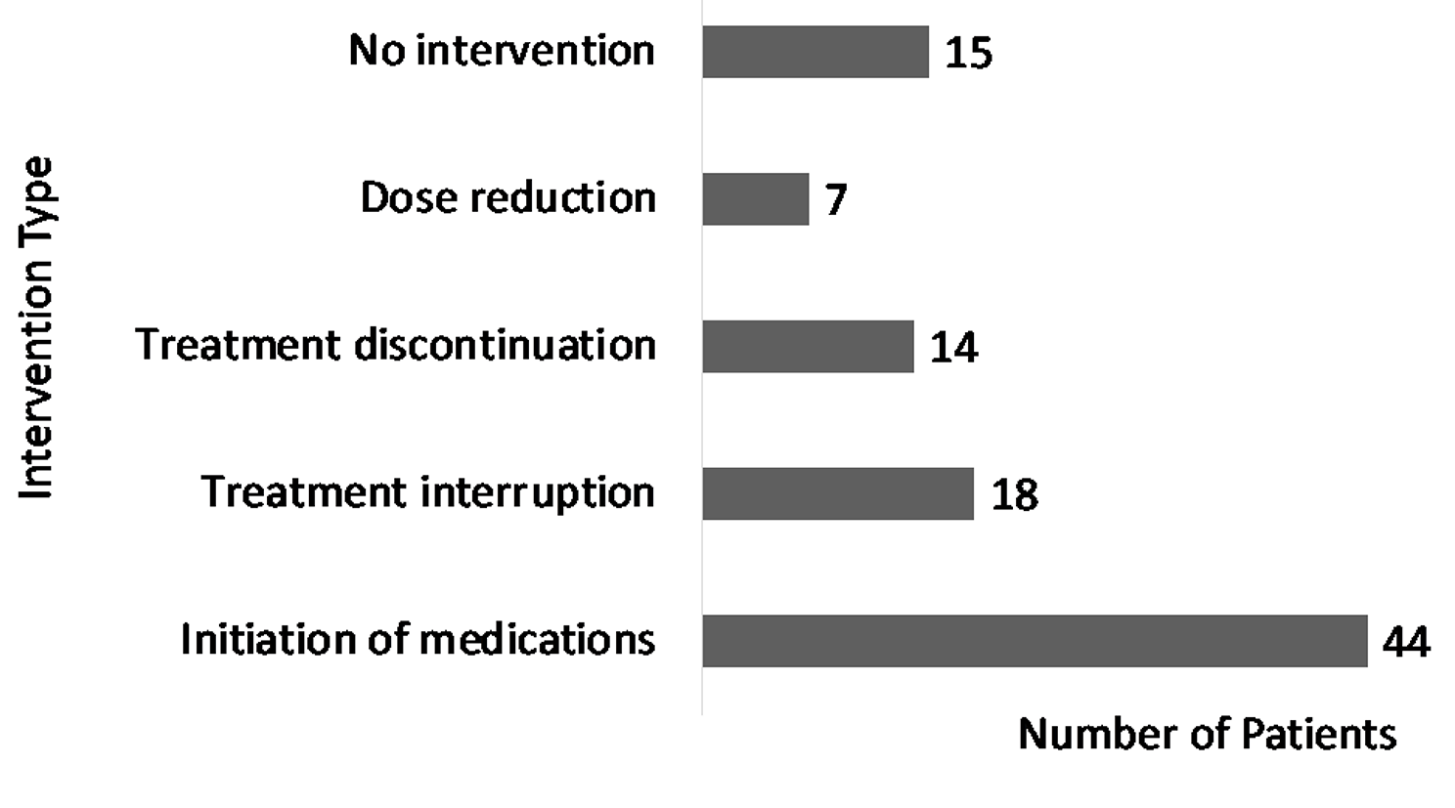
Characterization of cardio-oncologic interventions

The identified CV adverse events were HF/cardiomyopathy (31 events), arrhythmias (30 events), acute coronary syndromes (ACS) (14 events), pericardial disease (5 events), and deep vein thrombosis (DVT) (5 events). The three most common classes of oncolytic agents associated with HF were AIs (10 events), VEGFR inhibitors (6 events), and BCR-ABL inhibitors (6 events). With comparison to all reported events in the FAERS database, a significant ROR for HF and cardiomyopathy was found with AIs (ROR 2.45 [95% CI 2.31, 2.61], *p* < 0.0001) (Table 2). The median time to onset of CV adverse events ranged from 29 to 343 days and was shortest for pericardial disease and longest for DVT (Fig. 2). At presentation, 31 patients (46.2%) had elevated troponins. CV adverse events were managed by initiation of cardioprotective medications (*n* = 44), treatment interruption (*n* = 18), treatment discontinuation (*n* = 14), and/or dose reductions (*n* = 7). In 15 (22.4%) patients, no particular intervention was identified (Fig. 3). Upon presentation with HF, LVEF was ≤40% (heart failure with reduced ejection fraction or HFrEF) in 14 patients (45.2%), between 41 and 49% (heart failure with mildly reduced ejection fraction or HFmrEF) in 5 patients (16.1%), and ≥ 50% (heart failure with preserved ejection fraction or HFpEF) in 10 patients (32.3%). Among the 19 (61.3%) HFrEF or HFmrEF cases, angiotensin-converting enzyme inhibitors (ACEIs)/angiotensin receptor blockers (ARBs)/ angiontensin receptor neprilysin inhibitor(ARNI), beta blockers, and mineralocorticoid receptor antagonists (MRAs), were initiated in 15 (78.9%), 13 (68.4%), and 4 (21%) patients, respectively. Among 12 (38.7%) HFpEF cases, ACEI/ARB/ARNI, beta blockers, and MRAs, were initiated in 6 (50%), 7 (58.3%), and 1 (8.3%) patients, respectively. Sodium-glucose transport protein 2 inhibitors (SGLT2i) were not initiated in any HF patients.

**Table 2:** HF and cardiomyopathy events reported with AI and associated ROR

	HF Reactions	Other ADRs	ROR (95% CI) for AIs vs. full database, p value
AIs	1084	49,229	2.45 (2.31–2.61), *p* < 0.0001
Other drugs	215,754	24,036,165	

HF: Heart failure; ROR: Reporting odds ratio; AI: Aromatase inhibitor; ADR: Adverse drug reaction; CI: Confidence interval

Monitoring strategies were also described for patients who experienced HF since that was the largest subpopulation (Table 3). A follow-up echocardiogram (ECHO) was performed in 18 out of 31 patients at a median of 89 days (Interquartile range [IQR] 32.25-188.25). LVEF at follow-up was ≤40% in 8 patients (25.8%), between 41 and 49% in 5 patients (6.5%), ≥ 50% in 8 patients (25.8%). Out of the 14 patients who initially had a reduced EF (≤40%), 11 had available follow-up ECHOs, and LVEF was improved to ≥ 40% in 5 patients. Two patients who initially had mildly reduced LVEF experienced worsened LVEF to < 40% upon follow-up.

**Table 3:** Monitoring of Patients Experiencing HF (*N* = 31)

Ejection Fraction	Presentation N (%)	Follow-Up N (%)
≤40%	14 (45.2)	8 (25.8)
41–49%	5 (16.1)	2 (6.5)
≥ 50%	10 (32.3)	8 (25.8)
Not Available	2 (6.5)	13 (41.9)
Median time to follow-up (days)	89 (IQR 32.25-188.25)	
**BNP**		
Elevated BNP, N (%)	24 (77.4)	
Median BNP at presentation (pg/mL)	486 (IQR 208–957)	
Median pre-discharge BNP (pg/mL)^*^	340 (IQR 147.5–774)	
Median time to follow-up BNP (days)^**^	49 (IQR 23–130)	
Median BNP at follow-up (pg/mL)	412 (IQR 202–836)	

*Available for 21 (67.7%) patients

** Available for 24 (77.4%) patients

HF: Heart failure; IQR: Interquartile range

Fourteen (45.2%) patients presented with elevated troponins upon diagnosis of HF. B-type natriuretic peptide (BNP), a marker of cardiac stretch, injury, and/or increased filling pressures, was elevated in 77.4% of patients at presentation with a median of 486 pg/mL (IQR 208–957). A pre-discharge BNP was obtained in 21 (67.7%) patients and found to be decreased to a median of 340 pg/mL (IQR 147.5–774). A follow-up BNP was obtained in 24 (77.4%) patients after a median of 49 days (IQR 23–130). The median BNP at follow-up was slightly higher than the pre-discharge BNP with a median of 412 pg/mL (IQR 202–836).

## Discussion

In this cohort of 67 patients, HF was the most common CV adverse event with 31 events occurring at a median of 148 days (IQR 43–476). Roughly a third (32.2%) of the HF/cardiomyopathy events occurred in patients taking AIs. Of note, three prior meta-analyses of randomized controlled trials (RCTs) have shown an increased risk of CVD, mainly ischemic events, with AIs compared with tamoxifen, especially upon longer exposure [27–30]. The antioxidant and cardioprotective effects of tamoxifen have been attributed to its relatively lower risk of association with CV adverse events in comparison with AIs [29–31]. In a population-based cohort study, AIs were associated with an 86% increased risk of HF and a 50% increased risk of CV mortality compared with tamoxifen [30]. Another retrospective cohort study of 13,273 women with no history of CVD identified an increased risk of dysrhythmia, valvular dysfunction, and pericarditis with AIs vs. tamoxifen (adjusted HR, 1.29 [1.11–1.50]) as well as non-hormone users (1.18 [1.02–1.35]) [32]. The incidence of serious cardiac events (2.1% vs. 1.1%), including ischemic heart disease and HF, as well as hypercholesterolemia (43.6% vs. 19.2%) was significantly more common with letrozole compared with tamoxifen in the BIG (Breast International Group) 1–98 trial [33]. In the ATAC (Arimidex, Tamoxifen Alone or in Combination) trial, patients with pre-existing ischemic heart disease experienced more ischemic CV events (17% vs. 10%) when treated with anastrozole rather than tamoxifen; in addition, a higher percentage of patients taking anastrozole experienced cholesterol level elevation (9% vs. 3.5%) than those taking tamoxifen [34, 35]. In a recent matched-control study, breast cancer survivors treated with endocrine therapy appeared to have a higher risk of hypertension and diabetes [36]. The aforementioned association with ischemic heart disease and cardiometabolic risk factors could explain the indirect mechanism through which AIs may lead to HF. Of note, patients with a history of breast cancer on AIs may have a history of trastuzumab, anthracyclines, or chest radiation which can independently induce CV toxicity and act as a confounder. Out of the 10 patients observed to have received AIs and subsequently developed HF, none had received trastuzumab, 2 patients had a history of chest radiation and anthracycline use, and 1 patient had a history of chest radiation alone. To further investigate the association of AIs with HF, a signal disproportionality analysis was conducted based on a query of the FAERS pharmacovigilance database and revealed a significant association of AIs with HF (ROR 2.45 [95% CI 2.31, 2.61], *p* < 0.0001). One limitation of this query is that it does not capture asymptomatic CTRCD based on a new decline in GLS or rise in cardiac biomarkers. This perceived association of AIs with HF and/or cardiomyopathy bolsters prior findings and warrants further investigation in prospective studies for AIs’ association with both symptomatic and asymptomatic CTRCD.

Unlike AIs, HF has been well-described in other classes, and monitoring recommendations have been suggested. The recent ESC guidelines recommend a transthoracic echocardiogram (TTE) prior to initiation of VEGFR inhibitors, BTK inhibitors, RAF and MEK inhibitors in patients at high and very high risk. On the other hand, a TTE is recommended for all patients prior to initiation of second and third generation BCR-ABL inhibitors and osimertinib [20]. For example, a meta-analysis of 21 trials in different solid tumors that reported data on congestive HF (CHF) showed that subjects in the VEGFR TKI group were at significantly higher risk for CHF than subjects in the non-TKI group (RR = 2.69, *p* < 0.001, 95% CI: 0.1.86 to 3.87). However, a sub-analysis did not show an association of CHF with longer duration of therapy (≥ 17.2 weeks vs. <17.2 weeks) [37]. Similarly in this cohort study, the time to onset of HF varied widely indicating that a strong consideration should be made for the initial periodic monitoring to be extended for the duration of therapy. In fact, ESC guidelines suggest repeating TTE every 4 months for moderate-risk patients and every 3 months for high-risk patients during the first year of treatment with VEGFR inhibitors; following the first year, echocardiography every 6–12 months may be considered for both patient populations [20]. As previously described in the literature, individual VEGFR inhibitors appear to have different propensities for CV toxicity. A Bayesian network analysis of randomized controlled trials of nine VEGFR TKIs showed that lenvatinib and vandetanib tend to induce the most severe cardiotoxic manifestations while regorafenib and nintedanib did not display detectable increased risk of CV toxicity. Such comparisons of class agents are useful for clinicians to understand individual risks associated with these oral oncolytic agents and provide a framework to guide selection of treatment [38]. 

The BCR-ABL inhibitor which has strongly been associated with CV side effects is the third-generation agent ponatinib. In one retrospective study of 78 patients, the median time to onset of CV adverse events was 5.7 months, which is shorter than the reported 13.4 months for an arterial occlusive event in the PACE trial [39, 40]. While patients in the study did not develop peripheral or cerebrovascular occlusive events, which take longer to develop as shown in the PACE trial, the faster onset could also be related to a higher risk population in the real-world setting rather than the clinical trial setting. Interestingly, dasatinib was the most frequent BCR-ABL inhibitor associated with HF in this study. Historically, dasatinib carries the highest risk for immune-mediated pleural effusion and pulmonary hypertension. Because of the latter, a low threshold for performing an ECHO in patients with cardiopulmonary symptoms who will be started on dasatinib has been suggested [41]. In addition, ESC guidelines suggest that TTE should be considered every 3 months for high-risk patients during the first year of treatment with ponatinib or dasatinib; following the first year, echocardiography may be considered every 6–12 months [20]. 

Most studies of oral oncolytics excluded patients with low EF or other severe cardiovascular co-morbidities within 6 to 12 months of enrollment. In this cohort, 97% of the patients had pre-existing CVD or at least one risk factor for CVD [42, 43]. The results of this study highlight the fact that such patients are at an elevated risk for CV adverse events and should be monitored closely. Moreover, the results of this study add to the body of literature emphasizing the need to monitor patients on oral oncolytics closely and proactively manage any pre-identified CV risk factors. Pre-treatment risk assessment using recognized risk assessment tools such as Heart Failure Association–International Cardio-Oncology Society (HFA-ICOS) baseline cardiovascular toxicity risk assessment tool are recommended. Patients deemed to be at moderate-to-high risk would benefit from close surveillance, strict management of traditional CV risk factors, and a cardio-oncology referral. The ultimate risk represents a combination of patient-related risk factors and drug-related risk factors such as the incidence and severity of the associated CV toxicity [20]. Additional real-world studies on individual classes of medications can further quantify the risk with each class and help guide decision-making. For example, the FDA prescribing information for anastrozole was revised to include a warning for increased incidence of ischemic cardiovascular events in women with pre-existing CVD which was seen in the ATAC trial [35]. The current study is consistent with previous studies that indicate that the increased CV risk warning should be applicable to all AIs. General recommendations aimed at mitigating the risk associated with modifiable risk factors, such as smoking cessation, weight loss, increased physical activity, and pharmacologic interventions including lipid-lowering, anti-hypertensive, and anti-diabetic therapy, intuitively apply to the oncology population as well [42, 43]. Specific cardio-preventive interventions that have been tested in RCTs include ACEIs/ARBs, beta-blockers, or their combination and have yielded modest or no effect in attenuating LVEF or GLS decline. Additionally, such interventions have mainly been studied in patients on anthracyclines or trastuzumab [44]. Two meta-analyses have pooled studies evaluating neurohormonal inhibition and/or beta blockers in patients receiving anthracyclines and/or trastuzumab. Both analyses demonstrated that beta blockers and ACEI/ARBs may mitigate the decline in LVEF during trastuzumab and anthracycline treatments; however, the absolute benefit may be small with uncertain clinical relevance, and results may be skewed due to attrition and publication bias [45, 46]. Unfortunately, no studies on preventive cardioprotective strategies have yet been performed for attenuation of CV toxicity with oral oncolytics. In the current study, 32 (47.8%) and 28 (41.8%) patients were on beta blockers and ACEI/ARBs upon initiation of oral oncolytic therapy, respectively. Of the patients who did develop HF, 14 (45.2%) and 13 (41.9%) patients were on beta blockers and ACEI/ARBs upon initiation of oral oncolytic therapy, respectively. The development of CV adverse events in these patients despite early cardioprotective strategies underscores the high-risk nature of the cohort and reinforces the need for more effective preventive strategies that are validated with oral oncolytics.

Previous studies have suggested that troponin release pattern following high-dose chemotherapy may be utilized for the prediction of future ventricular dysfunction. In one study, prolonged troponin I elevation one month after therapy was associated with a higher decrease in LVEF [47]. Other small studies have also shown that serial troponin tests, alone or in combination with GLS measurement, may be beneficial for early detection of CV toxicity [48, 49]. To that end, high-sensitivity troponin assays for individual risk stratification and long-term risk prediction in the setting of stable coronary artery disease and heart failure are currently being investigated [50]. Based on previous study findings, risk-adapted strategies utilizing troponin as a surrogate endpoint were developed. The initial study by Cardinale et al. showed that, in patients treated with high-dose chemotherapy and deemed to be at high risk due to increased troponin I value, enalapril may prevent the development of late CV toxicity [51]. Subsequently, the open-label ICOS-ONE study found no difference in LVEF with a preventive strategy compared with troponin-triggered strategy for initiating enalapril in patients treated with anthracyclines [52]. In the current study, 14(45.2%) patients presented with elevated troponins upon identification of HF. Therefore, troponins could also be explored as a potential biomarker for CV toxicity secondary to oral oncolytics. The 2022 AHA/ACC AHA/ACC/HFSA Guideline for the Management of Heart Failure suggests serial measurement of cardiac troponin for risk stratification in patients receiving potentially cardiotoxic cancer treatments [53]. In addition, patients at risk of developing HF, BNP or NT-proBNP–based screening followed by collaborative care can be useful to prevent the development of LV dysfunction, diastolic dysfunction or new-onset HF [54]. The 2022 ESC cardio-oncology guidelines similarly recommend baseline measurement of NP and troponin for risk stratification and if the degree of change in the biomarkers will be used to detect subclinical cardiac injury during cancer treatment [20]. 

The paucity of literature describing whether the offending agent can be continued following development of CV toxicity complicates determining the optimal approach. The 2022 AHA/ACC AHA/ACC/HFSA Guideline for the Management of Heart Failure recommends discontinuing cardiotoxic therapy in patients who develop overt HF while a diagnostic workup is undertaken to establish the etiology and initiate guideline-directed medical therapy (GDMT) [53]. The guideline also promotes a collaborative, patient-centered approach, including a CV specialist in cardio-oncology and primary oncologist, when determining whether to resume, modify, or permanently discontinue therapy. The severity of HF, potential reversibility based on mechanism of toxicity, response to GDMT, and availability of alternative oncolytics can aid in decision-making. To date, there is no guidance for discontinuation or resumption of therapy with other types of cardiotoxicities. Based on the observed real-world cardio-oncologic interventions in this study, treatment was notably continued in 80% of the cases. Treatment was discontinued solely due to CV toxicity in only 3 cases. The most common causes for discontinuation included transition to hospice or death as well as progression shortly after development of the CV adverse events. In addition, pharmacological treatment of the CV adverse events was initiated in 65% of the cases. Oral oncolytics have been associated with high response rates and survival benefit in many instances, and reluctance to immediately withdraw these highly effective medications before attempting to manage their toxicities is justifiable. Furthermore, interruption of anti-HER2 agents because of CV adverse events was found to be associated with worse outcomes in patients with breast cancer [55, 56]. The aforementioned observation led to the emergence of the concept of permissive cardiotoxicity, which favors aggressive management of cardiotoxicity to enable the patient to remain on life-prolonging cancer treatment rather than discontinuation of cardiotoxic treatment [57]. For example, it is now recommended to continue anti-HER2 agents with close surveillance when CTRCD is asymptomatic and moderate [20]. In the oncology population, available data in patients with anthracycline and trastuzumab-induced HF suggest beta blockers and ACEi are effective in improving LV dysfunction [53]. Given the dearth of data specific to CTCRD secondary to oral oncolytics, management should align with the HF management guidelines. The AHA/ACC guideline recommends 4 medication classes for HFrEF stages C and D: beta blockers, ACEI/ARB/ARNi, MRA, and sodium/glucose cotransporter-2 inhibitors (SGLT2i). The same classes of medications may be considered in patients with HFmrEF. For patients with HFpEF, SGLT2i, MRAs, ARBs, and ARNi may be considered to decrease HF hospitalizations. SGLT2i is the only class of medications that has shown significant benefit in reducing CV mortality [58]. In the current study, adherence to GDMT was suboptimal; therefore, referral to a cardio-oncology service may help optimize treatment and outcomes. While the numbers are small, more patients had improved EF upon follow-up. Patients with HFrEF who improve their LVEF to > 40% are considered to have HFimpEF and should continue HFrEF treatment [53]. In most of these patients, cardiac structural abnormalities usually persist and may lead to an eventual decline in EF with non-adherence to the GDMT or re-introduction of cardiotoxic treatment [59]. 

**Fig. 4:**
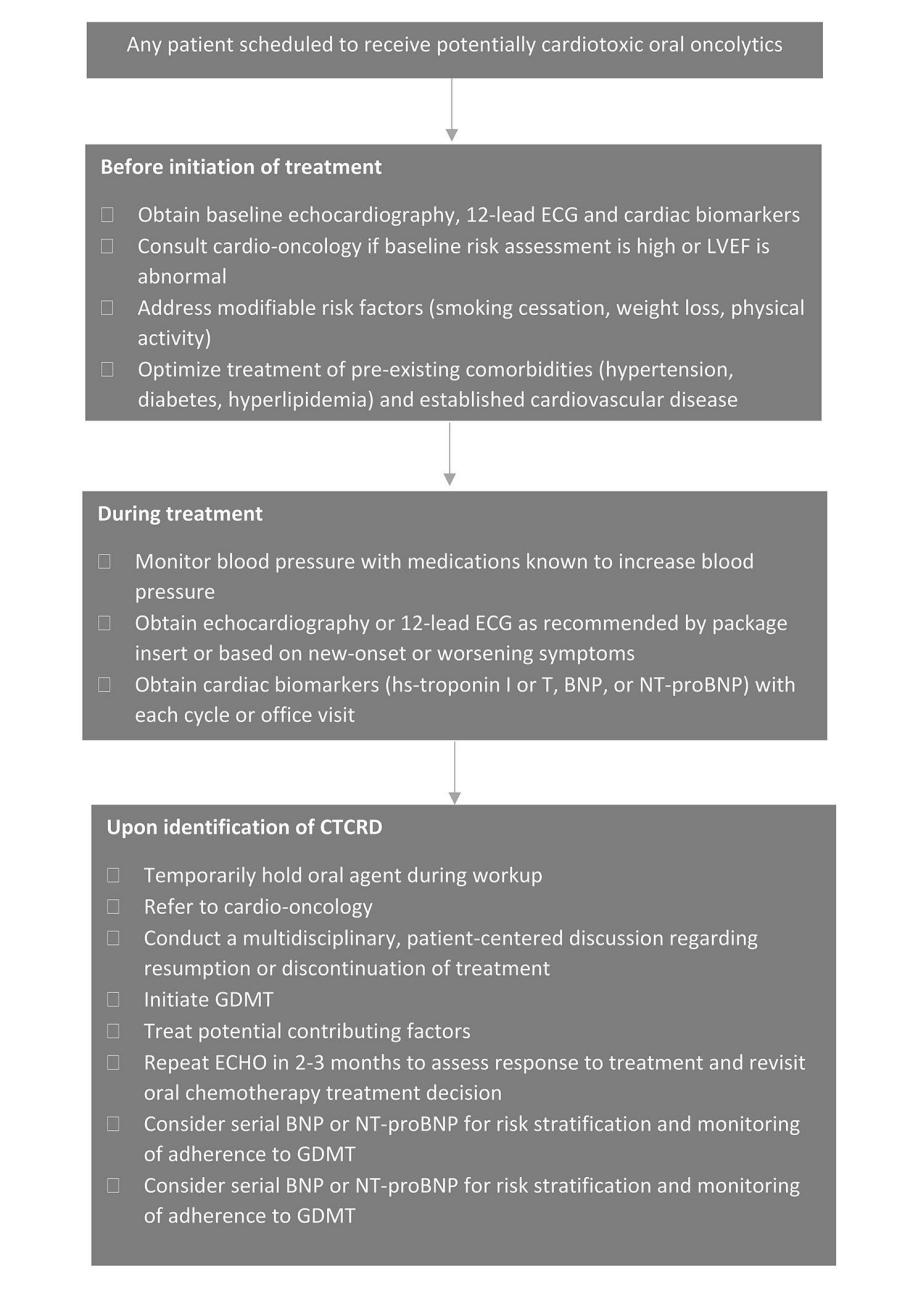
Algorithm for monitoring and treating patients for oral chemotherapy-induced cancer-therapy-related cardiac dysfunction (CTRCD)

In patients hospitalized for HF, measurement of BNP or NT-proBNP levels at admission is recommended as higher levels of BNP and NT-proBNP are associated with adverse short and long-term prognosis. A pre-discharge BNP or NT-proBNP level can also predict the disease course of the patient and establish a post-discharge prognosis [60, 61]. In the current cohort, median BNP at discharge was < 350 ng/L, which could signify a more favorable prognosis for death or readmission [62]. The median BNP in this study was not significantly changed after 2 months, possibly due to suboptimal adherence to GDMT. A proposed algorithm for monitoring and management of patients who present with CTCRD associated with oral oncolytics is outlined in Fig. 4.

Our study was primarily limited by the small population size which did not allow description of medication class-specific cardiotoxicities and consequent interventions. Our health-system is located in a large medical center. Our data pull identified outpatient prescriptions of patients who were treated at our health-system for any reason. The 1003 patients that were excluded were being treated for their cancer diagnosis by another health-system or private physician office, and as such, were not evaluable by chart review. The small sample size also prohibited regression analysis to investigate correlation of individual risk factors with the incidence of CV adverse events. Identification of CV adverse events also depended on reliability of accurate ICD-10 documentation and may have led to omission of eligible cases or misclassification of cardiotoxicities, although the latter was minimized by chart review. The observational and non-controlled nature of this study can only show association of oral oncolytics with CV adverse events and precludes ascertaining causality of agents in inducing CV toxicity. In addition, results may have been biased by the fact that the multi-health care hospital system has a large breast cancer center, and long-term survivors are seen frequently as part of routine surveillance. Data collection occurred primarily through retrospective manual chart review, which is limited by the variability in providers’ documentation and is prone to user error. Lastly, the short duration of follow-up does not allow assessment of the trajectory of CV adverse events or incidence of late CV adverse effects.

## Conclusion

Oral oncolytics are associated with different cardiotoxic manifestations, mainly CTRCD. Optimization of CV risk factors and comorbidities is crucial at all stages of cancer treatment. A multidisciplinary approach to treatment that includes appropriate monitoring of imaging and cardiac biomarkers, temporary interruption of treatment for CV adverse events, and initiation of guideline-directed treatment is recommended. The severity of CV adverse events, potential reversibility of suspected CV toxicity, response to pharmacological treatment, and availability of alternative treatment should be considered in the decision to resume or discontinue treatment. High-quality, drug-specific studies and cost-effectiveness analyses for screening and monitoring are also required for different classes of medications.

## Data Availability

The data that support the findings of this study are available from the corresponding author on reasonable request.

## Abbreviations

ACS: Acute coronary syndromes
ACEIs: Angiotensin-converting enzyme inhibitors
AIs: Aromatase inhibitors
ARBs: Angiotensin receptor blockers
ARNI: Angiontensin receptor neprilysin inhibitors
BCR-ABL: Breakpoint cluster region-Abelson murine leukemia
BMI: Body-mass index
BNP: B-type natriuretic peptide
CV: Cardiovascular
CVD: Cardiovascular disease
CTRCD: Cancer-therapy-related cardiac dysfunction
DVT: Deep vein thrombosis
ECHO: Echocardiogram
EGFR: Epidermal growth factor receptor
EMR: Electronic Medical Records
ESC: European Society of Cardiology
FAERS: FDA Event Reporting System
FDA: Food and Drug Administration
GDMT: Guideline-directed medical therapy
HER2: Human epidermal growth factor receptor 2
HF: Heart failure
HFimpEF: Heart failure with improved ejection fraction
HFmrEF: Heart failure with mildly reduced ejection fraction
HFpEF: Heart failure with preserved ejection fraction
HFrEF: Heart failure with reduced ejection fraction
IRB: Institutional review board
LVEF: Left ventricular ejection fraction
MRAs: Mineralocorticoid receptor antagonists
RCTs: Randomized controlled trials
ROR: Reporting odds ratio
SGLT2i: Sodium-glucose transport protein 2 inhibitors
TKI: Tyrosine kinase inhibitors
TTE: Transthoracic echocardiogram
VEGFR: Vascular endothelial growth factor receptor
